# Associations of Embeddedness and Posttraumatic Stress Disorder among 9/11 Survivors

**DOI:** 10.3390/epidemiologia2040041

**Published:** 2021-12-08

**Authors:** Meghan K. Hamwey, Cristina D. Pollari, Sukhminder Osahan, Sascha K. Garrey, Felix M. Ortega, Adrienne Solomon, Robert M. Brackbill

**Affiliations:** World Trade Center Health Registry, New York City Department of Health and Mental Hygiene, Long Island City, NY 11101, USA; cpollari@health.nyc.gov (C.D.P.); osukhmin@health.nyc.gov (S.O.); sgarrey@health.nyc.gov (S.K.G.); fortega@health.nyc.gov (F.M.O.); asolomon1@health.nyc.gov (A.S.); rbrackbi@health.nyc.gov (R.M.B.)

**Keywords:** embeddedness, PTSD, 9/11, social support, self-efficacy, social integration

## Abstract

Following exposures to traumatic events on 9/11, survivors have reported heightened levels of posttraumatic stress disorder (PTSD). Multiple factors contribute to both the exacerbation and amelioration of PTSD symptoms, including social integration and support. This cross-sectional study aimed to understand and identify associations of embeddedness and psychosocial risk factors by PTSD status for survivors and first responders of 9/11. Results indicate that those with chronic PTSD had the lowest prevalence of both social and emotional embeddedness and many who reported no PTSD symptoms following 9/11 reported moderate levels of social and emotional embeddedness. Overall, our findings suggest those individuals who reported little to no PTSD also reported the most social/emotional embeddedness; whereas those individuals who report greater or chronic PTSD report the least social/emotional embeddedness. As such, it may be beneficial for clinicians across multiple care disciplines and contexts to consider and address the social lives and needs of those individuals experiencing symptoms of PTSD to ensure their emotional and physical needs are truly being met.

## 1. Introduction

Post-traumatic stress disorder (PTSD) is the most prevalent mental health outcome among individuals directly and indirectly exposed to the terrorist attacks on 11 September 2001 (9/11) [1,2,3], and particularly among those who experienced trauma (e.g., injury, loss of a loved one) [4,5,6]. There are multiple mechanisms associated with PTSD (e.g., emotional engagement, stress tolerance, extinction, negative post-traumatic experiences), most of which tend to be driven by one’s psychology neurobiology, epigenetics, and/or biology. One of the psychological implications of PTSD is the avoidance of participation in social life, such as going to work or engaging in leisure activities. As a result of PTSD-related social withdrawal, there is often an increased and problematic use of substances such as alcohol, illicit drugs, and other substances with psychoactive properties [6,7,8]. A lack of social support and social integration have also been shown to be related to long-term PTSD [6,9,10]. The amelioration of PTSD symptoms would thus be bolstered by increased social support and social integration, or perhaps therapeutic intervention.

Social isolation, or the lack of social contact via in person or other means, is a characteristic feature of, experiencing PTSD symptoms and it can detrimentally affect individuals of all ages emotionally, physically, and mentally [11]. It comprises an objective deficit in both the quality and quantity of social ties and interactions with other individuals or a person’s wider community [12]. Further, social isolation has been found to be associated with a number of negative health outcomes, including an increased risk of mortality in older adults [13], depression [14], and suicidal ideation [15,16]. Loneliness, defined as the subjective feeling of a discrepancy between one’s desired and actual relationships, is an emotionally driven consequence of social isolation [17]. As with social isolation, loneliness is similarly linked to negative health outcomes, including mortality [18] and depression [18]. However, the literature is inconclusive on how the social dimensions of social isolation and the emotional dimensions of loneliness relate to their shared health risks. 

There is a growing body of literature that explores the concept of social and emotional embeddedness, which is the opposite of loneliness, in the context of individual demographic groups. For instance, Snowden and colleagues [19] found that, in a general sample of the United States population, African American men reported higher levels of overall social embeddedness than White men and that social involvement was an important factor in predicting psychological well-being. Another study that considered older Chinese adults living in rural areas, found that social embeddedness was positively associated with health information and help-seeking behavior via its status as a bolsterer of health-sharing behavior in this population [20]. Embeddedness further reflects both the size of social ties one has within their family and the greater community, as well as how connected one feels within their social network, irrespective of its size [21,22].

The presence or development of embeddedness could positively influence the course of PTSD. One factor that may contribute to greater embeddedness among individuals experiencing PTSD symptoms is individual self-efficacy, which signifies an individual’s belief in their personal capacity to perform in specific manners (e.g., social interactions). For instance, higher levels of coping self-efficacy have been found to have a buffering effect on PTSD symptoms and promote PTSD recovery [23,24]. Social integration, or the presence of social ties that can provide help and support to an individual’s perceived social support, or the subjective belief of how integrated one is in their social network, have also been found to be associated with lower levels of PTSD symptomology that result after exposure to natural disasters [25] and as potential sources for reduced stress among veterans [10]. In turn, we postulate that social and emotional embeddedness would have an association with the persistence of PTSD over time. Studies have categorized the course of PTSD since the 9/11-initiating event into trajectories that include resistant, delayed onset, recovering, or chronic [26]. Trajectories that remain high or chronic, that are refractory to the improvement of symptoms, are associated with low social support and 9/11-related unemployment [27]. It could be surmised that increased social and emotional embeddedness would lessen the persistence of PTSD and perhaps increase the effectiveness of treatment or be a treatment goal.

### Study Aims

Data from the World Trade Center Health Registry were used to explore the relationship between social and emotional embeddedness and the course of PTSD among persons directly exposed to the 9/11 attacks. We aim to: (1) describe the prevalence of social and emotional embeddedness among persons directly exposed to the 9/11 attacks, 15 years after their exposure, across four PTSD groups (i.e., no PTSD or resistant, delayed, recovered, and chronic), and (2) examine the associations of demographic factors and psychosocial characteristics (i.e., social support, social integration, and self-efficacy) with a new calculation of social and emotional embeddedness, stratified by probable 9/11-related PTSD status.

## 2. Materials and Methods

The World Trade Center Health Registry (WTCHR) was established by the New York City Department of Health and Mental Hygiene with the goal of monitoring the physical and mental health effects of those individuals exposed to the attacks on 9/11. The WTCHR is a longitudinal cohort study comprising 71,426 enrollees at the first survey wave (2003–2004). An additional three survey waves have also been collected in 2006–2007 (Wave 2), 2010–2011 (Wave 3), and 2015–2016 (Wave 4). The enrollees are comprised of rescue and recovery workers, lower Manhattan residents, local workers, school students and staff, and occupants/passersby on 9/11. Details of recruitment and data collection are published elsewhere [28].

The analytic sample for this study was restricted to enrollees who participated in the Health and Quality of Life (HQoL) 15 years after the 9/11 survey, which was an in-depth study of a subset of rescue and recovery workers and survivors who indicated they had sustained injuries on 9/11 and a comparison group of non-injured persons (*n* = 6544). The sample size for the study included all persons who reported being injured on 9/11/2001 and a 3 to 1 comparison group. Eligibility criteria included completing all four survey waves, being ≥18 years old at the time of collection, and English speaking. Enrollees who had missing data on the PTSD Checklist-Specific at any of the four survey waves were excluded from the study (*n* = 704). The final analytic sample resulted in 5840 enrollees. Importantly, the HQoL survey was only conducted at one time point, thus the present study is cross-sectional in design and analysis.

The WTCHR protocol was approved by the Institutional Review Boards at the Centers for Disease Control and Prevention and the New York City Department of Health and Mental Hygiene. Oral informed consent was obtained from participants at WTCHR enrollment using computer-assisted telephone interviews (CATI). This consent was in addition to the implicit consent by enrollees who completed each subsequent follow-up survey, as well as for the HQoL survey. Enrollees were regularly reminded by letter of their rights associated with being enrolled in the Registry, including the options of withdrawing from the Registry or not responding to follow-up surveys.

### 2.1. Measures

*Demographics*. Age collected at the time of the HQoL (ranges: <50, 50–59, 60–64, or ≥65 years old), sex collected at Wave 1 (male or female), race/ethnicity collected at Wave 1 (non-Hispanic White, non-Hispanic Black, Hispanic/Latino, Asian, or multiracial/other), Wave 4 employment (employed or not employed), and marital status (married/living with partner consistently across 4 survey waves, or not) were all included as covariates in this study.

Social and emotional embeddedness were measured using the De Jong Gierveld (2006) 6-item loneliness questionnaire [29] during the HQoL survey only and thus largely post-PTSD measures. The loneliness questionnaire contains three items specific to social loneliness and three for emotional loneliness. Item responses were categorical with three levels, “*yes*”, “*more or less*”, and “*no*”. Social and emotional loneliness scores were inverted to construct the embeddedness measure.

Social embeddedness. Social loneliness items were used to derive a social embeddedness construct. Items included “*There are plenty of people I can rely on when I have problems*”, “*There are many people I can trust completely*”, and “*There are enough people I feel close to*”. The social items were positively worded, and each response received the following reverse-scored value, yes = 0, more or less = 1, and no = 1. Items were summed to get a social loneliness total score, ranging from 0 to 3. Scores were not calculated when item responses were missing (*n* = 170). Social embeddedness was calculated by having a social loneliness total score of ≤1, that is those who indicated low levels of social loneliness were assigned a high level of embeddedness.

*Emotional embeddedness*. Emotional loneliness items were used to derive an emotional embeddedness construct. Items included “*I experience a general sense of emptiness*”, “*I miss having people around*”, and “*I often feel rejected*”. All emotional items were negatively worded and coded as *yes* = 1, *more or less* = 1, and *no* = 0 (Note that emotional and social embeddedness are reversed on direction of answer to questions). The items were summed to obtain a total emotional loneliness score, with scores ranging from 0 to 3. Scores were not calculated when item responses were missing (*n* = 174). Emotional embeddedness was calculated by having an emotional loneliness total score of ≤1. The thresholds for emotional and social embeddedness were derived from those used by De Jong Gierveld loneliness questionnaire scoring.

*Post-traumatic stress disorder*. Probable post-9/11 PTSD was evaluated across all four survey waves using the PTSD Checklist (PCL) 17-item questionnaire [30,31]. The PCL is a self-administered scale that assesses PTSD symptoms based on *DSM-IV* criteria [32]. Items focused on re-experiencing symptoms specific to 9/11 in the past 30 days, with responses ranging from 1 = *not at all* to 5 = *extremely*. A threshold of a PCL total score of 44 or greater indicated probable PTSD with a reported sensitivity of 0.78 and a specificity of 0.97 (see Ruggiero et al.).

Using the PCL total score, probable PTSD was organized into four different categories: *chronic*, *delayed*, *recovered*, and *no PTSD*. Those with no PTSD (resilient) were defined as having PCL total scores <44 across all 4 survey waves. Chronic PTSD was defined as having PCL total scores ≥44 across all 4 survey waves. Delayed PTSD was considered as having PCL total scores <44 at Waves 1 and 2 and PCL total scores ≥44 at Waves 3 and 4. Lastly, the recovered group was expressed as PCL total scores ≥44 at Waves 1 and 2 and PCL total scores <44 at Waves 3 and 4.

*Social integration*. Social integration was measured using four items adapted from the RAND Social Health Battery [33]. Participants were asked to indicate whether they have experienced the following in the last 30 days: “*having 1 or more close friends*”, “*having visited/talked to/emailed friends at least twice in the last 30 days*”, “*attended a religious service at least twice in last 30 days*”, or “*been actively involved in a volunteer organization or club in last 30 days*”. The number of endorsed items were summed and categorized using three levels: *low* (0–1), *medium* (2), and *high* (≥3). Prior use of these categories of social integration provided a clear dose–response relationship with various outcomes including depression.

*Social support*. Social support was measured at Wave 4 using the Social Support Survey for the Medical Outcomes Study, 5-item version [34]. Items asked participants how often someone was available to perform the following tasks for them: “*have a good time with*”, “*hug you*”, “*take you to the doctor*”, “*prepare your meals if you are unable*”, and “*understand your problems*”. Responses ranged from 0 = *none of the time* to 4 = *all of the time*. The five items were summed with total scores ranging from 0-20. Social support was categorized as *low* ≤10, *medium* 11–15, and *high* as 16–20, similarly to social integration categories.

*Self-efficacy*. Self-efficacy was collected at Wave 4 using the General Self-Efficacy Scale (GSE) [35], which included five items measured on a four-point scale. The items were “*It is easy for me to stick to my aims and accomplish my goals*”, “*I am confident that I could deal efficiently with unexpected events*”, “*Thanks to my resourcefulness, I know how to handle unforeseen situations*”, “*I can remain calm when facing difficulties because I can rely on my coping abilities*”, and “*No matter what comes my way, I am usually able to handle it*”. Responses ranged from 0 = *not at all true* to 4 = *exactly true*. Items were summed to create a total score ranging from 0-20. *Low self-efficacy* was defined as having a total score <17 and *high self-efficacy* as ≥17, based on the median score for the entire Registry sample.

### 2.2. Analytic Strategy

Overall frequencies of demographic characteristics and psycho-social variables were calculated. Bivariate analyses were conducted for demographic characteristics and psycho-social variables with social and emotional embeddedness, using unadjusted odds ratios and 95% confidence intervals.

Multivariable and Log-linear regression models were used to examine the association of the psycho-social variables with social and emotional embeddedness, separately. Each model was adjusted for age, race, sex, and stratified by PTSD group (chronic, delayed, recovered, no PTSD). Odds ratios and 95% confidence intervals were computed. The significance level was set at a 2-sided value of alpha < 0.05 to identify plausible relationships between PTSD history and social and emotional embeddedness recognizing that multiple comparisons may yield associations by chance that are not statistically significant at this level.

All analyses were conducted using SAS 9.4 (SAS Institute, Inc., Cary, North Carolina).

## 3. Results

### 3.1. Demographic Characteristics

A total of 5840 enrollees were included in this sample. On average, enrollees were 58 years old (*M* = 58.1; *SD* = 10.2). The majority of enrollees were male (64.9%) and White (78.6%). Further, the majority of enrollees indicated they were employed (57.5%) and married/living with a partner (58.7%) (not shown in tables).

### 3.2. Social Embeddedness

Table 1 shows the distribution of demographic factors and psycho-social factors with social embeddedness, stratified by PTSD group. The majority of enrollees never experienced PTSD (*n* = 4097) with a smaller portion falling into the other three PTSD groups. The delayed PTSD group was comprised of 309 enrollees, 406 were chronic, and 97 recovered over time. Overall, social embeddedness was more common among females (46.3%), those younger than 50 years of age (49.0%), those who were consistently married (49.9%), non-Hispanic Whites (47.9%), and those who indicated they were employed (46.7%). Further, social embeddedness was more frequently reported among those with greater levels of social support (63.1%), social integration (52.3%), and self-efficacy (58.1%). The highest prevalence of social embeddedness was found in the no PTSD group (52.2%), followed by the recovered (32.0%), delayed, (26.2%), and chronic (16.0%) groups.

The multivariable logistic regression results of the psycho-social factors with social embeddedness, stratified by PTSD group are presented in Table 2. High social support was positively associated with social embeddedness, across all four PTSD groups. The no PTSD and chronic PTSD groups showed a statistically significant dose–response relationship with the varying levels of social support. High social integration was significantly associated with social embeddedness for those in the no PTSD (aOR = adjusted odds ratio; aOR = 2.23; 95% CI: 1.48, 3.36) and chronic PTSD (aOR = 4.93; 95% CI: 1.20, 20.26) groups. Social integration was not significantly associated in the delayed or recovered PTSD group. Those exhibiting higher levels of self-efficacy had 1.30 (95% CI: 1.22, 1.39) times the odds of experiencing social embeddedness in the no PTSD group. Self-efficacy was not statistically significant in the adjusted models for the delayed, chronic, or recovered PTSD groups.

### 3.3. Emotional Embeddedness

Table 3 shows the distribution of demographic factors and psycho-social factors with emotional embeddedness, stratified by PTSD group. Overall, emotional embeddedness was prevalent among males (69.6%), those aged 65 or older (71.6%), those who were consistently married (72.2%), non-Hispanic Whites (69.9%), those who indicated they were employed (70.8%), and those with greater levels of social support (82.6%), social integration (73.6%), and self-efficacy (83.0%). The prevalence of emotional embeddedness varied across the PTSD groups. The no PTSD group had the highest prevalence (68.0%), followed by the recovered (41.7%), delayed (36.5%), and chronic group (25.7%).

Table 4 presents the multivariable logistic regression results of the psycho-social factors with emotional embeddedness by each PTSD group. Social support showed a dose-response relationship with medium and high levels increasing the odds of emotional embeddedness in the no PTSD, delayed, and recovered groups. Social support was not statistically significant among those in the chronic PTSD group. High social integration was significantly associated with emotional embeddedness in the no PTSD (aOR = 1.35; 95% CI: 1.11, 1.63) group. High self-efficacy was also significantly associated with emotional embeddedness in the no PTSD (aOR = 1.21; 95% CI: 1.17, 1.25) and chronic (aOR = 1.62; 95% CI: 1.12, 2.35) groups. Social integration and self-efficacy were not associated with emotional embeddedness in the delayed or recovered groups.

## 4. Discussion

As was hypothesized, persons who had chronic PTSD had the lowest prevalence of both social and emotional embeddedness and over half of persons with no symptoms indicative of PTSD following 9/11 reported social and emotional embeddedness. Only a slightly higher proportion of those who “recovered” indicated social and emotional embeddedness than the delayed group. Surprisingly, the prevalence of emotional embeddedness was higher than social embeddedness for all PTSD strata, suggesting the importance of addressing the emotional needs of 9/11 survivors, in addition to their social needs. More nuanced findings for PTSD status were found, suggesting that social embeddedness was greater among the no PTSD and chronic PTSD status, and social integration was higher for the recovered or delayed PTSD status.

Critically, research tends to focus on the social aspects of loneliness or isolation and how these factors contribute to one’s well-being [36,37,38], while gerontological studies tend to focus on the social connections individuals develop and maintain during their later years and how their presence contributes to their health [39,40,41]. Further, the associations between loneliness and isolation, and their antithesis of embeddedness, have not previously been demonstrated with regard to the potential associations with PTSD. Given the abundance of literature on PTSD and its potential correlates for physical health functioning among trauma-exposed populations this was a particularly important facet of this study, which is the first to address the concepts of emotional and social embeddedness and their associations with PTSD among a 9/11-exposed population to our knowledge.

Findings from this study suggest that individuals who reported experiencing no symptoms of PTSD reported greater emotional and social embeddedness, whereas those who reported symptoms of PTSD, whether chronic, delayed, or recovered, were less likely to do so. Thus, these findings further support the notion that the social and emotional barriers often coupled with symptoms of PTSD are likely to detrimentally influence one’s ability and willingness to engage in socially or emotionally embedding situations (e.g., attend social events). Through identifying these connections, this study helps to better expand on potential factors that may ultimately improve social and emotional functioning for those individuals experiencing symptoms of PTSD (e.g., social activities, emotional connections/supports). Among those enrollees who ever experienced PTSD symptoms, we found that the recovered group tended to report the highest levels of social and emotional embeddedness, followed by the delayed group, with the lowest prevalence reported by the chronic group. Moreover, it appears that the prevalence of social embeddedness was less among those individuals who experienced PTSD symptoms, compared with emotional embeddedness, which could be attributed to a variety of factors including socio-demographic, neighborhood/community, access, and focus of treatment. Further, it is possible that emotional embeddedness in contrast to social embeddedness may play a more prominent role in abating symptoms of PTSD among those who experienced or continue to experience PTSD after an exposure to a traumatic event. Importantly, future research would need to evaluate whether social or emotional embeddedness precedes or is a result of a reduction in PTSD symptoms. Critically, we must keep in mind that regardless of PTSD status, the experiences of embeddedness, and the alternative, loneliness, are subjective and are hard to measure and track over time [42]. It is also possible that those enrollees who reported delayed or chronic PTSD may have experienced additional traumas post-9/11 that were not captured in this study. Specifically, data on traumas post-9/11 were only captured during Wave 4 and when included in our models, did not change the strength of the associations.

Another explanation of less social than emotional embeddedness among persons in the PTSD recovery group is that social embeddedness implies an outward nature of acting or engaging with others. On the one hand, those who did not report experiencing any PTSD symptoms would be more likely to report social embeddedness given the lack of psychological barriers associated with PTSD. On the other hand, individuals who report PTSD symptoms may experience varying degrees of psychological barriers, in turn impacting the ability to engage in more socially driven activities. As evidenced in a sample of Vietnam veterans, those with chronic PTSD may become more accustomed to the nature of the disorder and choose to avoid social situations in order to reduce the likelihood of being triggered [43]. As such, future studies may benefit from considering whether PTSD symptom clusters among those who express greater avoidance symptoms would, in fact, report diminished embeddedness.

Other factors may also contribute to the experience of social embeddedness among the recovered group. For instance, individuals who no longer experience symptoms of PTSD may have received additional therapeutic interventions that increased their feelings of self-efficacy, in turn encouraging them to engage in more socially oriented ways. In fact, among persons with no PTSD symptoms who reported higher levels of self-efficacy, we found they were 1.3 times as likely to report social embeddedness compared with those in the delayed, recovered, and chronic PTSD group (see Table 3). This suggests that the mere presence of PTSD symptoms, whether enduring or not, may diminish one’s sense of self-efficacy, and in turn, reduce one’s ability to experience more social embeddedness. Garrey et al. [44] found that those individuals who sought treatment had greater self-efficacy than those not in treatment for PTSD. Conversely, it may also be that people with low self-efficacy could be more likely to experience PTSD.

As indicated, all four of the groups in this study reported a higher prevalence of emotional embeddedness compared with social embeddedness. One potential reason for this difference may be related to how emotional embeddedness reflects a more internal or intrinsic experience and perspective. As opposed to engaging with others socially, emotional embeddedness allows for individuals to become “close” to individuals without the need for physical proximity. In fact, studies suggest that the interactions one has with others may be less critical for developing meaningful relationships; rather, the quality of the relationship and emotional connection may be more salient [45].

### Limitations

Despite the many strengths of this study, several limitations must be mentioned. First, it is important to note that although we attempted to include as many enrollees as possible in our analyses, our PTSD groups were not all equal in size. Due to this, it is possible that some of the smaller sample-sized PTSD groups were not as sufficiently powered and our ability to detect true effect sizes was diminished. Importantly, these sample size differences are typically the product of enrollees being lost to follow-up and not to other factors within the control of the researchers. Further, although the de Jong Gierveld 6-item loneliness measure [28] used in this study has been validated in a previous study, our specific scoring strategies and usage of the scale as a reverse measure of embeddedness has not been validated. Future studies should attempt to replicate these findings using these measurement strategies to validate the findings and confirm their usage in this manner. As mentioned in the discussion, individuals who reported delayed or chronic PTSD may have done so due to additional traumatic experiences they had post-9/11. Given our study only captured additional traumas post-9/11 at one time point, it would be beneficial for future studies to gather this information at multiple time points to assess the impact of traumatic exposure, as well as control for extraneous impacts, including history effects, post-9/11. It may also be beneficial to expand upon the quantitative data collection strategies by incorporating a qualitative design to better understand the underlying factors driving feelings or experiences of embeddedness. Critically, given this survey was conducted 15 years post-9/11, the causal implications of PTSD and embeddedness are not able to be determined. Despite our efforts to gather PTSD information across all Waves of the Registry surveys, the HQoL survey was only conducted once. As such, it may be that embeddedness may potentially be a consequence of PTSD, as opposed to a driving mechanism. Future studies would benefit from addressing this potential additional consideration, as well as gathering embeddedness information over time. Finally, although the Registry possesses many benefits it is important to note that it is a closed cohort of individuals with data collection periods often spanning large intervals of time. Therefore, it is difficult for us to fully determine the timing of PTSD group membership and when/if movement from one group to another has occurred. Future research would benefit from further addressing these PTSD groupings to determine the static and dynamic nature of their classifications and what factors, including those of emotional and social embeddedness, may contribute to or thwart these transitions.

## 5. Conclusions

This study assessed the prevalence and association of social embeddedness and psychosocial factors by PTSD status for survivors and first responders of 9/11. Our findings suggest that those individuals who report little to no PTSD also report the most social/emotional embeddedness; whereas those individuals who report greater or chronic PTSD report the least social/emotional embeddedness. To better address the nature of social and emotional embeddedness among those individuals who report symptoms of PTSD, clinicians across professional contexts should address the social lives of these individuals and ensure their emotional and physical needs are being met.

## Figures and Tables

**Table 1:** Distribution of demographic factors and psycho-social factors with social embeddedness.

	All four PTSD Groups			No PTSD Group			Delayed PTSD Group			Chronic PTSD Group			Recovered PTSD Group		
	No	Yes	Total	No	Yes	Total	No	Yes	Total	No	Yes	Total	No	Yes	Total
	*n* = 3100	*n* = 2570	5670	*n* = 1959	*n* = 2138	4097	*n* = 228	*n* = 81	309	*n* = 341	*n* = 65	406	*n* = 66	*n* = 31	97
Characteristic	54.7%	45.3%		47.8%	52.2%		73.8%	26.2%		84.0%	16.0%		68.0%	32.0%	
Gender															
Male	2036	1651	3687	1313	1402	2715	158	59	217	198	32	230	38	17	55
	55.2	44.8		48.4	51.6		72.8	27.2		86.1	13.9		69.1	30.9	
Female	1064	919	1983	646	736	1382	70	22	92	143	33	176	28	14	42
	53.7	46.3		46.7	53.3		76.1	23.9		81.3	18.8		66.7	33.3	
Age on Injury Survey (years)															
<50	597	573	1170	388	480	868	48	24	72	53	8	61	12	5	17
	51.0	49.0		44.7	55.3		66.7	33.3		86.9	13.1		70.6	29.4	
50–59	1075	791	1866	640	644	1284	98	25	123	134	28	162	24	13	37
	57.6	42.4		49.8	50.2		79.7	20.3		82.7	17.3		64.9	35.1	
60–64	607	437	1044	372	356	728	42	15	57	68	10	78	19	6	25
	58.1	41.9		51.1	48.9		73.7	26.3		87.2	12.8		76.0	24.0	
65+	821	769	1590	559	658	1217	40	17	57	86	19	10	11	7	18
	51.6	48.4		45.9	54.1		70.2	29.8		81.9	18.1		61.1	38.9	
Marital Status W1–W4															
Always	1665	1658	3323	1101	1414	2515	121	50	171	150	38	188	30	17	47
	50.1	49.9		43.8	56.2		70.8	29.2		79.8	20.2		63.8	36.2	
Never/sometimes	1435	912	2347	858	724	1582	107	31	138	191	27	218	36	14	50
	61.1	38.9		54.2	45.8		77.5	22.5		87.6	12.4		72.0	28.0	
Race/ethnicity															
Non-Hispanic White	2323	2139	4462	1527	1799	3326	166	71	237	217	44	261	44	25	69
	52.1	47.9		45.9	54.1		70.0	30.0		83.1	16.9		63.8	36.2	
Non-Hispanic Black	261	143	404	149	117	266	23	0	23	39	10	49	5	0	5
	64.6	35.4		56.0	44.0		100.0	0.0		79.6	20.4		100.0	0.0	
Hispanic	300	170	470	154	121	275	25	7	32	50	9	59	12	6	18
	63.8	36.2		56.0	44.0		78.1	21.9		84.8	15.3		66.7	33.3	
Asian	117	73	190	73	64	137	6	2	8	19	1	20	2	0	2
	61.6	38.4		53.3	46.7		75.0	25.0		95.0	5.0		100.0	0.0	
Multiracial/other	99	45	144	56	37	93	8	1	9	16	1	17	3	0	3
	68.8	31.3		60.2	39.8		88.9	11.1		94.1	5.9		100.0	0.0	
Employment W4															
No	1365	1048	2413	777	823	1600	110	45	155	217	39	256	23	14	37
	56.6	43.4		48.6	51.4		71.0	29.0		84.8	15.2		62.2	37.8	
Yes	1735	1522	3257	1182	1315	2497	118	36	154	124	26	150	43	17	60
	53.3	46.7		47.3	52.7		76.6	23.4		82.7	17.3		71.7	28.3	
Social Support W4															
Low	1027	219	1246	511	144	655	98	16	114	188	15	203	32	4	36
	82.4	17.6		78.0	22.0		86.0	14.0		92.6	7.4		88.9	11.1	
Medium	1025	604	1629	657	495	1152	85	21	106	94	21	115	22	8	30
	62.9	37.1		57.0	43.0		80.2	19.8		81.7	18.3		73.3	26.7	
High	998	1704	2702	759	1465	2224	42	43	85	51	28	79	12	19	31
	36.9	63.1		34.1	65.9		49.4	50.6		64.6	35.4		38.7	61.3	
Social Integration W4															
low 0–1	200	35	235	81	17	98	23	3	26	54	2	56	3	2	5
	85.1	14.9		82.7	17.4		88.5	11.5		96.4	3.6		60.0	40.0	
Medium 2	1379	875	2254	858	723	1581	93	28	121	157	21	178	28	11	39
	61.2	38.8		54.3	45.7		76.9	23.1		88.2	11.8		71.8	28.2	
High 3–4	1477	1616	3093	988	1363	2351	109	48	157	127	41	168	35	18	53
	47.8	52.3		42.0	58.0		69.4	30.6		75.6	24.4		66.0	34.0	
Self-efficacy W4															
low <17	1944	983	2927	1042	728	1770	180	49	229	291	49	340	48	15	63
	66.4	33.6		58.9	41.1		78.6	21.4		85.6	14.4		76.2	23.8	
High 17+	1134	1570	2704	905	1396	2301	44	31	75	46	16	62	18	16	34
	41.9	58.1		39.3	60.7		58.7	41.3		74.2	25.8		52.9	47.1	

**Table 2:** Multivariable logistic regression results of the psycho-social factors with social embeddedness.

	All four PTSD Groups			No PTSD Group			Delayed PTSD Group			Chronic PTSD Group			Recovered PTSD Group		
	aOR	95% CI		aOR	95% CI		aOR	95% CI		aOR	95% CI		aOR	95% CI	
Characteristic		Lower	Upper		Lower	Upper		Lower	Upper		Lower	Upper		Lower	Upper
Marital Status W1-W4															
Always	0.99	0.94	1.06	0.99	0.93	1.06	1.01	0.68	1.51	0.81	0.50	1.29	1.25	0.68	2.28
Never/sometimes	ref			ref						ref					
Employment W4															
No	ref														
Yes	**1.06**	**1.01**	**1.12**	1.05	1.00	1.12	0.95	0.66	1.38	1.05	0.61	1.47	0.68	0.40	1.15
Social Support W4															
Low	ref			ref			ref			ref			ref		
Medium	**1.95**	**1.70**	**2.24**	**1.86**	**1.59**	**2.19**	1.30	0.69	2.45	**2.27**	**1.18**	**4.36**	2.87	0.91	9.08
High	**2.99**	**2.63**	**3.41**	**2.68**	**2.30**	**3.12**	**3.07**	**1.77**	**5.35**	**3.91**	**2.11**	**7.23**	**4.99**	**1.77**	**14.03**
Social Integration W4															
Low 0–1	ref			ref			ref			ref			ref		
Medium 2	**1.68**	**1.25**	**2.26**	**1.89**	**1.25**	**2.84**	1.35	0.42	4.28	2.46	0.57	10.59	0.57	0.13	2.47
High 3–4	**2.04**	**1.52**	**2.73**	**2.23**	**1.48**	**3.36**	1.52	0.49	4.67	**4.93**	**1.20**	**20.26**	0.72	0.16	3.16
Self-efficacy W4															
Low <17	ref			ref			ref			ref			ref		
High 17+	**1.39**	**1.31**	**1.48**	**1.30**	**1.22**	**1.39**	1.33	0.94	1.90	1.45	0.86	2.45	1.52	0.85	2.73

Note: All models are adjusted for age, sex, and race/ethnicity; Bold estimates are statistically significant at the 0.05 level. Ref = reference; aOR = adjusted odds ratio; 95% CI = 95% confidence interval.

**Table 3:** Distribution of demographic factors and psycho-social factors with emotional embeddedness, stratified by PTSD group.

	All four PTSD Groups			No PTSD Group			Delayed PTSD Group			Chronic PTSD Group			Recovered PTSD Group		
	No	Yes	Total	No	Yes	Total	No	Yes	Total	No	Yes	Total	No	Yes	Total
	*n* = 1815	*n* = 3851	5666	*n* = 857	*n* = 3239	4096	*n* = 197	*n* = 113	310	*n* = 298	*n* = 103	401	*n* = 56	*n* = 40	96
Characteristic	32.0%	68.0%		32.0%	68.0%		63.6%	36.5%		74.3%	25.7%		58.3%	41.7%	
Gender															
Male	1118	2562	3680	521	2190	2711	138	79	217	172	56	228	31	22	53
	30.4	69.6		19.2	80.8		63.6	36.4		75.4	24.6		58.5	41.5	
Female	697	1289	1986	336	1049	1385	59	34	93	126	47	173	25	18	43
	35.1	64.9		24.3	75.7		63.4	36.6		72.8	27.2		58.1	41.9	
Age on Injury Survey(years)															
<50	359	824	1183	177	699	876	46	27	73	51	11	62	8	9	17
	30.4	69.7		20.2	79.8		63.0	37.0		82.3	17.7		47.1	52.9	
50–59	649	1208	1857	291	987	1278	80	44	124	115	44	159	21	16	37
	35.0	65.1		22.8	77.2		64.5	35.5		72.3	27.7		56.8	43.2	
60–64	358	688	1046	160	571	731	33	24	57	58	19	77	14	11	25
	34.2	65.8		21.9	78.1		57.9	42.1		75.3	24.7		56.0	44.0	
65+	449	1131	1580	229	982	1211	38	18	56	74	29	103	13	4	17
	28.4	71.6		18.9	81.1		67.9	32.1		71.8	28.2		76.5	23.5	
Marital Status W1–W4															
Always	926	2401	3327	452	2069	2521	109	64	173	142	43	185	23	22	45
	27.8	72.2		17.9	82.1		63.0	37.0		76.8	23.2		51.1	48.9	
Never/sometimes	889	1450	2339	405	1170	1575	88	49	137	156	60	216	33	18	51
	38.0	62.0		25.7	74.3		64.2	35.8		72.2	27.8		64.7	35.3	
Race/ethnicity															
Non-Hispanic White	1344	3120	4464	668	2660	3328	148	89	237	188	73	261	37	31	68
	30.1	69.9		20.1	79.9		62.5	37.6		72.0	28.0		54.4	45.6	
Non-Hispanic Black	138	262	400	48	216	264	17	6	23	37	11	48	4	1	5
	34.5	65.5		18.2	81.8		73.9	26.1		77.1	22.9		80.0	20.0	
Hispanic	203	266	469	80	194	274	21	12	33	44	13	57	11	7	18
	43.3	56.7		29.2	70.8		63.6	36.4		77.2	22.8		61.1	38.9	
Asian	70	120	190	34	103	137	4	4	8	17	3	20	1	1	2
	36.8	63.2		24.8	75.2		50.0	50.0		85.0	15.0		50.0	50.0	
Multiracial/other	60	83	143	27	66	93	7	2	9	12	3	15	3	0	3
	42.0	58.0		29.0	71.0		77.8	22.2		80.0	20.0		100.0	0.0	
Employment W4															
No	864	1541	2405	349	1249	1598	99	56	155	195	58	253	24	12	36
	35.9	64.1		21.8	78.2		63.9	36.1		77.1	22.9		66.7	33.3	
Yes	951	2310	3261	508	1990	2498	98	57	155	103	45	148	32	28	60
	29.2	70.8		20.3	79.7		63.2	36.8		69.6	30.4		53.3	46.7	
Social Support W4															
Low	749	499	1248	292	366	658	92	21	113	158	44	202	30	6	36
	60.0	40.0		44.4	55.6		81.4	18.6		78.2	21.8		83.3	16.7	
Medium	556	1068	1624	277	873	1150	59	47	106	80	33	113	14	15	29
	34.2	65.8		24.1	75.9		55.7	44.3		70.8	29.2		48.3	51.7	
High	469	2232	2701	266	1958	2224	43	44	87	51	25	76	11	19	30
	17.4	82.6		12.0	88.0		49.4	50.6		67.1	32.9		36.7	63.3	
Social Integration W4															
Low 0–1	158	75	233	47	50	97	22	4	26	49	6	55	3	2	5
	67.8	32.2		48.5	51.6		84.6	15.4		89.1	10.9		60.0	40.0	
Medium 2	814	1434	2248	389	1189	1578	81	41	122	131	46	177	22	15	37
	36.2	63.8		24.7	75.4		66.4	33.6		74.0	26.0		59.5	40.5	
High 3–4	817	2280	3097	407	1947	2354	90	67	157	116	49	165	31	23	54
	26.4	73.6		17.3	82.7		57.3	42.7		70.3	29.7		57.4	42.6	
Self-efficacy W4															
Low <17)	1337	1585	2922	560	1206	1766	151	78	229	259	79	338	41	22	63
	45.8	54.2		31.7	68.3		65.9	34.1		76.6	23.4		65.1	34.9	
High 17+	460	2246	2706	288	2017	2305	42	34	76	35	24	59	15	18	33
	17.0	83.0		12.5	87.5		55.3	44.7		59.3	40.7		45.5	55.5	

**Table 4:** Multivariable logistic regression results of the psycho-social factors with emotional embeddedness, stratified by PTSD group.

	All Four PTSD Groups			No PTSD Group			Delayed PTSD Group			Chronic PTSD Group			Recovered PTSD Group		
	aOR	95% CI		aOR	95% CI		aOR	95% CI		aOR	95% CI		aOR	95% CI	
Characteristic		Lower	Upper		Lower	Upper		Lower	Upper		Lower	Upper		Lower	Upper
Marital Status W1–W4															
Always	1.00	0.96	1.04	0.99	0.96	1.03	1.25	0.93	1.68	1.33	0.93	1.92	1.05	0.68	1.63
Never/sometimes	ref			ref			ref			ref			ref		
Employment W4															
No	ref			ref			ref			ref			ref		
Yes	**1.09**	**1.05**	**1.13**	1.03	0.99	1.06	1.06	0.80	1.40	1.18	0.85	1.65	1.32	0.78	2.24
Social Support W4															
Low	ref			ref			ref			ref			ref		
Medium	**1.55**	**1.44**	**1.67**	**1.33**	**1.23**	**1.43**	**2.63**	**1.67**	**4.15**	**1.32**	**0.88**	**1.97**	**3.34**	**1.49**	**7.49**
High	**1.78**	**1.66**	**1.92**	**1.47**	**1.37**	**1.58**	**2.88**	**1.82**	**4.54**	**1.42**	**0.92**	**2.20**	**3.62**	**1.60**	**8.20**
Social Integration W4															
Low 0–1	ref			ref			ref			ref			ref		
Medium 2	**1.53**	**1.28**	**1.84**	**1.27**	**1.05**	**1.54**	1.92	0.74	4.99	1.80	0.80	4.07	1.11	0.38	3.21
High 3–4	**1.66**	**1.38**	**1.99**	**1.35**	**1.11**	**1.63**	2.04	0.80	5.19	2.10	0.93	4.73	1.17	0.41	3.32
Self-efficacy W4															
Low <17	ref			ref			ref			ref			ref		
High 17+	**1.35**	**1.30**	**1.40**	**1.21**	**1.17**	**1.25**	1.16	0.86	1.56	**1.62**	**1.12**	**2.35**	1.20	0.72	2.01

Note: All models are adjusted for age, sex, and race/ethnicity; bold estimates are statistically significant at the 0.05 level. Ref = reference; aOR = adjusted odds ratio; 95% CI = 95% confidence interval.

## Data Availability

This data is not publicly available. Requests for this or other Registry data can be sent to rbrackbi@health.nyc.gov.
